# Cancer data quality and harmonization in Europe: the experience of the BENCHISTA Project – international benchmarking of childhood cancer survival by stage

**DOI:** 10.3389/fonc.2023.1232451

**Published:** 2023-08-22

**Authors:** Angela Lopez-Cortes, Fabio Didonè, Laura Botta, Lisa L. Hjalgrim, Zsuzsanna Jakab, Adela Cañete Nieto, Charles Stiller, Bernward Zeller, Gemma Gatta, Kathy Pritchard-Jones, The BENCHISTA Project Working Group, Joanne Aitken

**Affiliations:** ^1^ University College London (UCL) Great Ormond Street Institute of Child Health, Developmental Biology & Cancer Research Department, London, United Kingdom; ^2^ Fondazione IRCCS “Istituto Nazionale dei Tumori di Milano” (INT), Department of Evaluative Epidemiology, Milan, Italy; ^3^ BENCHISTA Project Management Team, London, United Kingdom

**Keywords:** childhood cancer, population-based, cancer registry, Toronto staging, diagnosis, survival, data quality, data harmonization

## Abstract

**Introduction:**

Variation in stage at diagnosis of childhood cancers (CC) may explain differences in survival rates observed across geographical regions. The BENCHISTA project aims to understand these differences and to encourage the application of the Toronto Staging Guidelines (TG) by Population-Based Cancer Registries (PBCRs) to the most common solid paediatric cancers.

**Methods:**

PBCRs within and outside Europe were invited to participate and identify all cases of Neuroblastoma, Wilms Tumour, Medulloblastoma, Ewing Sarcoma, Rhabdomyosarcoma and Osteosarcoma diagnosed in a consecutive three-year period (2014-2017) and apply TG at diagnosis. Other non-stage prognostic factors, treatment, progression/recurrence, and cause of death information were collected as optional variables. A minimum of three-year follow-up was required. To standardise TG application by PBCRs, on-line workshops led by six tumour-specific clinical experts were held. To understand the role of data availability and quality, a survey focused on data collection/sharing processes and a quality assurance exercise were generated. To support data harmonization and query resolution a dedicated email and a question-and-answers bank were created.

**Results:**

67 PBCRs from 28 countries participated and provided a maximally de-personalized, patient-level dataset. For 26 PBCRs, data format and ethical approval obtained by the two sponsoring institutions (UCL and INT) was sufficient for data sharing. 41 participating PBCRs required a Data Transfer Agreement (DTA) to comply with data protection regulations. Due to heterogeneity found in legal aspects, 18 months were spent on finalizing the DTA. The data collection survey was answered by 68 respondents from 63 PBCRs; 44% of them confirmed the ability to re-consult a clinician in cases where stage ascertainment was difficult/uncertain. Of the total participating PBCRs, 75% completed the staging quality assurance exercise, with a median correct answer proportion of 92% [range: 70% (rhabdomyosarcoma) to 100% (Wilms tumour)].

**Conclusion:**

Differences in interpretation and processes required to harmonize general data protection regulations across countries were encountered causing delays in data transfer. Despite challenges, the BENCHISTA Project has established a large collaboration between PBCRs and clinicians to collect detailed and standardised TG at a population-level enhancing the understanding of the reasons for variation in overall survival rates for CC, stimulate research and improve national/regional child health plans.

## 1 Introduction

According to the International Agency for Research of Cancer (IARC) and estimates from 2020, nearly 280.000 children and teenagers (0-19 years old) were diagnosed with cancer around the world and almost 110.000 died of this cause (1). When considering estimates of total childhood cancer incidence accounting for underdiagnosis, a simulation-based analysis found that there were 397.000 incident cases of childhood cancer for 200 territories worldwide and 43% of these were undiagnosed with substantial variation by region (range:3%-57%). Furthermore, considering population projections for 2015-2030 it is estimated there will be 6.7 million cases of CC worldwide, from which 2.9 million of cases will be missed (2). In addition to these estimates, and due to delay in diagnosis, variation in treatment and rates of relapse, paediatric oncology patients in low-and middle-income countries (LMICs) are five times as likely to die from a cancer diagnosis compared with patients in high-income countries (HICs) (3).

Population-based cancer registries (PBCRs) are key organizations that generate estimates of incidence and survival essential for cancer research (4). When considering paediatric patients, data completeness and accuracy represent a challenge due to the rarity and heterogeneity of childhood cancer. It has also been noted that stage data is not consistently recorded for paediatric patients. The tumour/node/metastasis (TNM) system is the standard staging system for most adult cancers; however, it is inappropriate for documenting the extent of disease in most childhood cancers (5).

Disease-specific staging systems have been developed for childhood cancers within the context of broader risk-stratification schemes used by various clinical trial groups. This means that for many diagnostic groups, two or more systems are in clinical use and there was no international standard suitable for global use by population-based cancer registries. Thus, in 2014 and through a collaborative effort between epidemiologists, clinical trial groups and registration experts, a consensus definition of tumour stage was agreed for most childhood cancer types - the Toronto Paediatric Cancer Stage Guidelines (TG) for population cancer registries (4, 6). These are endorsed by the European Network of Cancer Registries (ENCR), the Group for Cancer Epidemiology and Registration in Latin Language Countries (GRELL) the African Network of Cancer Registries (ANCR) and published in the UICC TNM Classification of Malignant Tumours 8^th^ Edition (5, 7).

Different childhood cancer population-based studies have demonstrated survival disparities between countries and European regions. Several factors may explain this variation including late diagnosis, delayed treatment, variation in quality of diagnostic and treatment services, management of acute complications, lack of resources, limited access to health services, abandonment to treatment, among others (8–10). Further understanding of the international variation in childhood cancer survival may be explained by the distribution of stage at diagnosis and stage-specific survival (4, 5, 11–13).

Other research studies have assessed the feasibility and validity of the TG demonstrating that PBCRs can reconstruct stage according to TG (11). This standardised framework supports PBCRs to assign cancer stage using data that can be found routinely in clinical records for most childhood cancers. The success of the pilot study emphasised the importance of a larger number of cancer registries in different countries applying the TG so that the paediatric staging system can be further improved (11, 14).

The International Benchmarking of Childhood Cancer Survival by Stage, also called BENCHISTA Project, is a research collaboration between multiple population-based cancer registries from European and non-European countries. It aims to stimulate the application of Toronto Stage Guidelines by participating PBCRs for six of the most common paediatric solid cancers (15) to lead to a better understanding of the reasons for variation in childhood cancer survival between countries and to highlight areas for improvement. The research sponsors are University College London (UCL) and the Fondazione IRCCS Istituto Nazionale dei Tumori di Milano (INT).

Due to the large number of participating PBCRs, data quality, harmonization and standardization are essential. The aim of this paper is to present the resources that the project established to ensure high-quality, standardised data, comparable across participating PBCR. The resources used have provided understanding on current procedures at cancer registry level and highlighted strengths and limitations when gathering stage at diagnosis, other prognostic, and non-stage prognostic factors to understand childhood cancer survival and its variation.

## 2 Materials and methods

All European population-based cancer registries (PBCRs) included in the EUROCARE studies were invited to participate in the BENCHISTA Project. Additionally, other non-European PBCRs from Australia, Canada, Brazil, and Japan confirmed their contribution to the project. A great number of PBCRs are checked for quality indicators by the International Agency for Research on Cancer (IARC) based on four dimensions of quality: comparability, validity, timeliness, and completeness (16, 17). Not all PBCRs have a government mandate. Some are coordinated by the National Society for Paediatric Haematology-Oncology and/or register all cases diagnosed at all hospitals authorised for childhood cancer treatment in the relevant country, with the aim of achieving population coverage.

Participating PBCRs were required to assign stage at diagnosis at a population-level using the Internationally recognized Toronto Stage Guidelines (TG) to six paediatric solid tumours (Ewing sarcoma, osteosarcoma, rhabdomyosarcoma – for ages 0 to 19 years old – and neuroblastoma, medulloblastoma and Wilms Tumour – for ages 0 to 14 years old) diagnosed in a three-year period (in the window 2014-2017). The selection of these tumour diagnoses was based on several factors, including their unambiguous diagnosis by histological code, previous studies showing geographical disparities in outcomes, limited improvements in survival rates over a prolonged period, and their significant representation among childhood solid tumours (4, 9, 10). For the three sarcomas, we included cases in the adolescent range (15 to 19 years old) as many of the participating registries collected data in this age range, where bone and soft tissues sarcomas peak in incidence. The process of determining the stage of diagnosis was performed by cancer registrars or relevant staff, who use various available data sources, such as clinical records, histopathology and imaging reports, and other administrative files. Clinical personnel could also be consulted in cases where uncertain or inconclusive information was encountered.

The staging classification used the TG to enable registries to derive the best estimate of stage at diagnosis in a standardised fashion. The guidelines endorse a two-tier approach, Tier 1 focuses on registries with limited resources and/or restricted data access and requires less detailed criteria and stage categories; Tier 2 involves more detailed criteria for cancer registries with further access to medical information or well-resourced (4, 5, 11). All Tier 2 can be converted into Tier 1. For the BENCHISTA Project, TG is defined as extent of disease at the time of diagnosis and based on detailed evidence before receiving treatment with two exceptions: staging of localized (non-metastatic) Wilms Tumour after neoadjuvant chemotherapy, since stage is based on surgical and histopathological examination of the nephrectomy specimen; and tumours in which investigations to exclude distant metastases may be performed shortly after surgery to the primary tumour but before systemic therapy is commenced.

The variables collected included depersonalized patient demographic data plus information on clinical investigations and types of data sources used by the registrars for applying Toronto staging for each of the six solid tumours (e.g., imaging/examinations performed and their results, when available). PBCRs were requested to use the International Classification of Childhood Cancers (ICCC-3) and to assign tumour stage according to the Toronto consensus staging guidelines. Moreover, to review all available data sources and to seek advice from clinicians when further explanation or clarification was required to ensure consistency in data collection and accuracy. Follow-up for life status was requested for a minimum of three years from diagnosis.

The BENCHISTA Project also assessed the availability to PBCRs of optional but clinically relevant variables for understanding any variation in treatment and survival for the six solid tumours. These optional variables included the more recently agreed ‘Toronto non-stage prognostic factors’ (NSP), and primary treatment modalities, relapse/recurrence/progression, and cause of death (6). To avoid limitations due to language barriers, the TG provided detailed guidance (5) translated in different languages (Italian, Spanish, Japanese, French, Bulgarian and Portuguese) and an electronic tool available to facilitate its use by different audiences (18, 19).

Data gathered from each participating PBCR were merged in a maximally anonymized dataset created by and stored within the secure environment of the data controller at INT. Comparative analysis of distribution of tumour stage at diagnosis at a population-level and analysis of survival estimates by stage for each tumour type between large geographical regions with similar groupings to previous EUROCARE studies is in progress. Validation is being conducted by the project analytical team to verify the coverage, number of submitted cases and national/local reported incidence.

Several factors were considered for standardization and harmonization parameters. Data files were checked with *ad hoc* developed procedures in regular use by the data controller (INT). Likewise, the validity of each variable and variable combinations for each tumour record were checked to detect unlikely or incorrect values. Records that were flagged during the data checking process were sent to the registries for revision and amendment. Furthermore, cases ascertained only by death certificate (DCO), number of cases diagnosed by cytology and those with unspecified morphology codes (NOS) were considered as data quality indicators for the completeness and accuracy of population-level data. Additional assessments to define the accuracy of sub-typing definitions in the six solid tumours of interest were also conducted.

To support the TG staging by PBCRs and ensure standardized processes, a series of three online training workshops led by clinical experts in the six solid tumours of interest and generated in collaboration with the Belgian Cancer registry were held. Moreover, to understand the modalities of data collection and staging processes in each PBCR a survey was designed to verify local/national processes and understand current practices and the possibility to seek advice from clinicians when clarification is required. This survey was addressed to registrars, clinical and non-clinical staff gathering data and completing stage at diagnosis for the BENCHISTA Project.

To assess data comparability, a quality assurance tool including a set of twelve fictitious cases (two per each tumour type of interest) was developed and completed by selected representatives of each participating cancer registry. Both surveys were developed using the platform SurveyMonkey^®^ and the results were gathered and analysed by the team at INT.

### 2.1 Project’s governance

The Project Management Team (PMT) comprises the two principal investigators in the UK and Italy, and four representatives from participating cancer registry staff (Norway, Denmark, Spain and Hungary). The Project Working Group (PWG) involves one or two representatives from each contributing cancer registry, six tumour-specific oncology experts nominated by the relevant European clinical study group, representatives from parent/survivor groups and communication and dissemination partners. The BENCHISTA team, PMT and PWG meet regularly to review the project’s advances and overview preliminary results helping to ensure a broader assessment of tasks and upcoming plans to guarantee the achievement of the project’s goals.

Moreover, the project has established an Independent Advisory Board (IAB) that includes a cancer registry director not directly involved in the day-to-day project, parent and survivor representatives, clinical executive level members of a national paediatric oncology society, a clinical trial study group and a medical director-level clinician involved in organisation of childhood cancer services. Importantly, there is also representation by patient/public involvement and engagement (PPIE) structures to ensure the perspective of parents and survivors is included in the different stages of the project’s development and upcoming results.

## 3 Results

### 3.1 PBCR participation and database status

**Figure 1:**
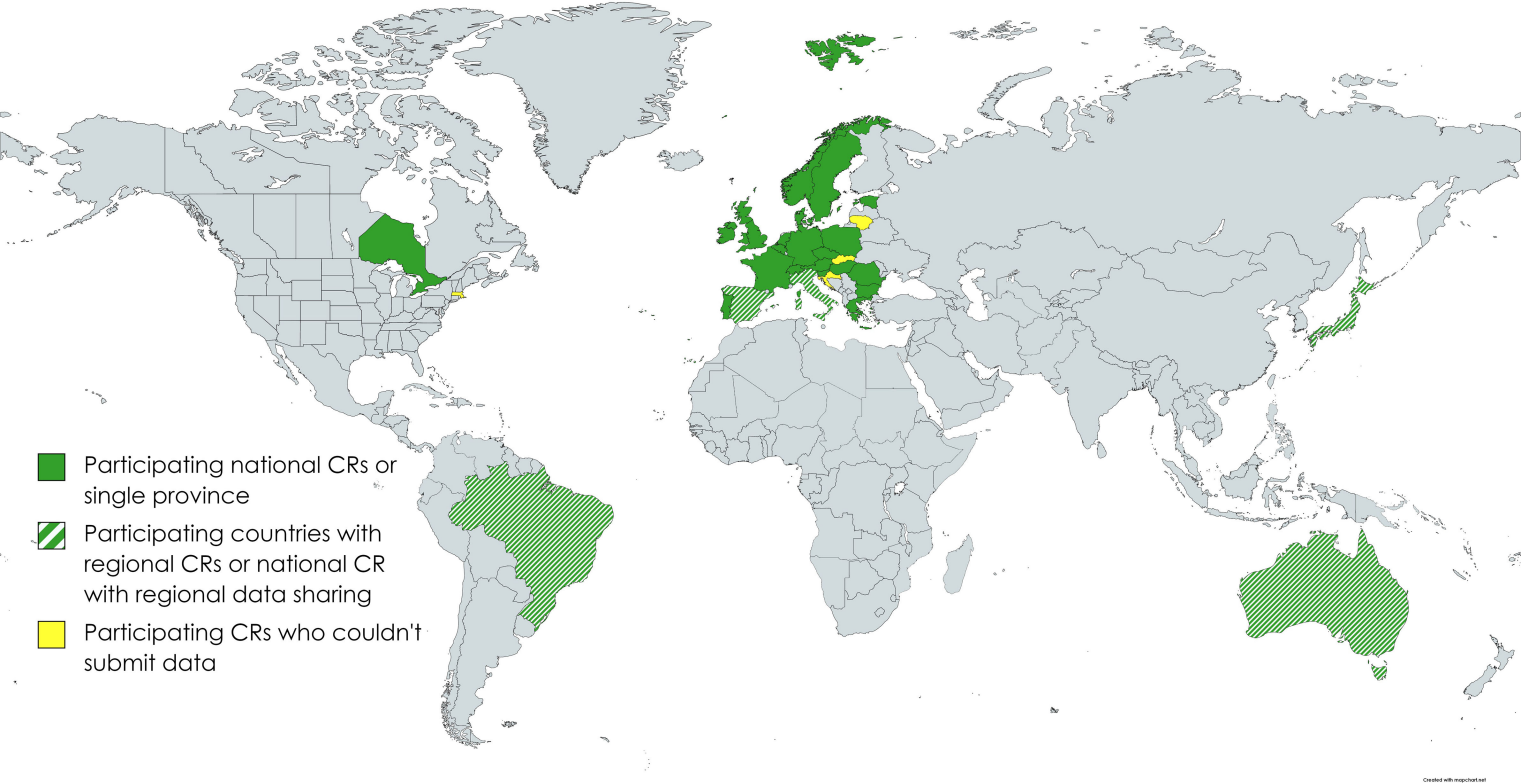
Participating countries.

80 PBCRs within and outside of Europe were interested to participate in the project at outset, however 16% of them could not participate due to different reasons, including limited or no availability of population-based data, anticipated restrictions in sharing patient-level datasets beyond national boundaries and limited access to clinical data to apply TG. 67 PBCRs from 24 European countries, Australia, Brazil, Japan, and Canada committed to participate in the project, which commenced in January 2021 (**Figure 1**). This process entailed close work between the data controller, cancer registry leaders and in some cases legal representatives from the PBCRs to achieve research collaboration.

Seeking for standardised TG collection by PBCRs, three on-line workshops were held in October-November 2021. A total of 60 PBCRs, both within and outside Europe, actively participated in real-time; each session attracted an attendance ranging from 70 to 80 individuals and was centred in two specific tumour types. The sessions covered various topics, including the fundamental principles of Toronto Staging, introducing and discussing clinical aspects, diagnosis, therapy, and non-stage prognostic factors for each tumour type and exemplar staging exercises based on pre-created cases. The training workshops were recorded and are publicly available on the BENCHISTA Project website, together with other supporting materials.

The content and format of compulsory and optional data variables were agreed by the Project Working Group members to ensure almost complete anonymisation whilst retaining patient-level information (**Table 1**). Although the process to agree the content of the required datafile submitted by each registry was finalised by March 2021, heterogeneity in the approach and legal requirements from participating countries led to a lengthy process to finalise the format and content of the data sharing agreement and hence delays in data submission.

**Table 1:** Variables and dataset structure.

Variable	No. of characters	Notes and encoding
Basic variables		
Registry	10	alphabetic
Registry Patient Identification code	10	assigned by the registry, it is a project-specific pseudonymised code
Year of birth	4	yyyy
Age at diagnosis	3	Numeric (in months)
Year of diagnosis	4	yyyy
Sex	1	boy/girl/unknown 1/2/9
Base of diagnosis (as coded in the ENCR protocol)	1	DCO/Clinical/Clinical investigation/Specific tumour markers/Cytology/Histology of a metastasis/Histology of a primary tumour/Unknown 0/1/2/4/5/6/7/9
ICDO-3-Topography	3	Only the numeric part of the ICD-O-3 topography code will be reported (the “C” and “.” will not be included)
ICDO-3-Morphology	4	Malignant, only, behaviour=3
First previous cancer	1	Y/N/unknown 1/0/9
First previous cancer definition		International Classification of Childhood Cancers (ICCC) 3rd edition
Year of diagnosis of the first previous cancer	4	yyyy/9
Second previous cancer	1	Y/N/unknown 1/0/9
Second previous cancer definition		International Classification of Childhood Cancers (ICCC) 3rd edition
Year of diagnosis of the second previous cancer	4	yyyy/9
Imaging/examination used for staging before any treatment		
CT/MRI primary site	1	Y/N/unknown 1/0/9
MRI whole neuraxis	1	Y/N/unknown 1/0/9
MRI whole neuraxis outcome		Negative/Positive/Suspicious/Unknown 0/1/2/9
CT thorax	1	Y/N/unknown 1/0/9
CT thorax outcome		Negative/Positive/Suspicious/Unknown 0/1/2/9
Imaging of regional lymph nodes	1	Y/N/unknown 1/0/9
Imaging of regional lymph nodes outcome		Negative/Positive/Suspicious/Unknown 0/1/2/9
CSF	1	Y/N/unknown 1/0/9
CSF outcome		Negative/Positive/Suspicious/Unknown 0/1/2/9
MIBG scan	1	Y/N/unknown 1/0/9
MIBG scan outcome		Negative/Positive/Suspicious/Unknown 0/1/2/9
Abdominal ultrasound	1	Y/N/unknown 1/0/9
Abdominal ultrasound outcome		Negative/Positive/Suspicious/Unknown 0/1/2/9
Bone scan	1	Y/N/unknown 1/0/9
Bone scan outcome		Negative/Positive/Suspicious/Unknown 0/1/2/9
Bone marrow aspirate or biopsy	1	Y/N/unknown 1/0/9
Bone marrow aspirate or biopsy outcome		Negative/Positive/Suspicious/Unknown 0/1/2/9
X-Ray thorax	1	Y/N/unknown 1/0/9
X-Ray thorax outcome		Negative/Positive/Suspicious/Unknown 0/1/2/9
PET	1	Y/N/unknown 1/0/9
PET outcome		Negative/Positive/Suspicious/Unknown 0/1/2/9
Tissue biopsy	1	Y/N/unknown 1/0/9
Tissue biopsy outcome		Negative/Positive/Suspicious/Unknown 0/1/2/9
Source used for staging		
Clinical report (hospital clinical records)	1	Y/N/unknown 1/0/9
Pathological report	1	Y/N/unknown 1/0/9
Administrative files (hospital discharge, etc.)	1	Y/N/unknown 1/0/9
Clinical study group	1	Y/N/unknown 1/0/9
Others (string)	10	alphabetic
Toronto staging, Neuroblastoma		
Stage Tier 1	2	L/LR/M/MS/X 1/2/3/4/9
Stage Tier 2	2	L1/L2/M/MS/X 1/2/3/4/9
Laterality	1	Not applicable/Right/Left/Unilateral NOS/Bilateral//unknown 0/1/2/3/4/9
* NSP: N-Myc	1	Amplified Y/N (exact definitions to be discussed)
Toronto staging, Wilms tumour		
Stage Tier 1 after pre-surgery chemotherapy	1	L/M/X 1/2/9
Stage Tier 2 after pre-surgery chemotherapy	1	y-I/y-II/y-III/IV/9 1/2/3/4/9
Stage Tier 1 after immediate surgery (i.e., surgery first)	1	L/M/X 1/2/9
Stage Tier 2 after immediate surgery	1	I/II/III/IV/X 1/2/3/4/9
Laterality	1	R/L/B 1/2/3
O_NSP: Wilms Presence of anaplasia	1	No/Yes, but unknown if focal or diffuse/Yes, focal/Yes, diffuse/Anaplasia unknown 0/1/2/3/9
Toronto staging, Medulloblastoma		
Stage Tier 1	1	L/M/X 1/2/9
Stage Tier 2	2	M0/M1/M2/M3/M4/X 0/1/2/3/4/9
*Evaluation of postoperative residual disease		R0/R1/R2/R+/unknown 0/1/2/3/9
*_NSP: Wingless (WNT) medulloblastoma	1	Y/N/unknown 1/0/9
*_NSP: Sonic Hedgehog (SHH) medulloblastoma	1	Y/N/unknown 1/0/9
Toronto staging, Osteosarcoma, Ewing sarcoma		
Stage Tier 1	1	L/M/X 1/2/9
Stage Tier 2	1	L/M/X 1/2/9
Toronto staging, Rhabdomyosarcoma		
Stage Tier 1	1	L/M/X 1/2/9
Stage Tier 2	1	I/II/III/IV/X 1/2/3/4/9
*_NSP: FKR-PAX3 rhabdomyosarcoma	1	Y/N/unknown 1/0/9
*_NSP : FKR-PAX7 rhabdomyosarcoma	1	Y/N/unknown 1/0/9
Primary Treatment defined as given within 1 year from diagnosis		
*_Surgery	1	Y/N/unknown 1/0/9
*_Chemotherapy	1	Y/N/unknown 1/0/9
*_Chemotherapy type	1	Preoperative chemo/Postoperative chemo/Both, preoperative and postoperative chemo/Chemotherapy only/Unknown 1/2/3/4/9
*_Radiotherapy	1	Y/N/unknown 1/0/9
*_Relapse/recurrence/progression		
*_Relapse/recurrence/progression	1	Y/N/unknown 1/0/9
*_Time in days from diagnosis to relapse/recurrence/progression		numeric
Follow-up		
Status of life alive/dead	1	alive/dead/unknown 1/2/9
*_Causes of death (CoD)	1	Toxicity of treatment, Tumour, Comorbidity previously present in the child, Others, unknown 1/2/3/4/9
Time in days from diagnosis to death or last follow up		numeric

*Optional Variables.

Data started to flow to the data controller from March 2022 and up to now 58 databases including nearly 11000 cases have been successfully submitted and are under final stages of quality assessment. Specific queries on data or further requests to ensure high-quality are discussed among the Project Management Team and the PBCR if required. Model answers to each query received were collated and published on the project website as a series of Frequently Asked Questions.

### 3.2 Data privacy process and challenges

While data sharing and data transfer involve movement of data from one institution to other, there are several differences between these two concepts. Data sharing involves making data available more broadly, enabling reuse and access in ways that allow control and management from one or several parties. Data transfer involves moving specific data from one entity to another with a purpose and typically to solve a specific research question; it tends to be more targeted and involves providing the information to another entity for analysis, storage but without giving full control over data itself. Both, data sharing, and data transfer are subjected to legal and ethical considerations that need to be considered to comply with General Data Protection Regulation principles including purpose minimization, lawfulness of processing, accuracy, storage limitation and accountability (20).

In compliance with institutional and legal requirements, the project was granted with ethical approvals from UCL and INT. Minor amendments to the protocol and appendix were submitted and approved to ensure information is clear and adequate. Individual parent consent is not required in general as the information is collected under existing permissions for cancer registration in each jurisdiction.

Each participating cancer registry was approached to understand their individual requirements to proceed with data sharing. For 26 PBCRs, the project-specific ethical approval obtained by the research sponsors (UCL and INT) was sufficient to confirm their participation and submit data for analysis. The rest of the PBCRs required a specific legal document that allowed collaboration and further research.

Considering requirements and aims of the project, a Data Transfer Agreement (DTA) was developed by the legal officer and data controller’s team at INT. It contains general information about the project and legal considerations to share patient-level data in a highly de-personalised format. Its aim is to meet specific legislations to adhere to General Data Protection Regulations (GDPR) and relevant laws from each participating country; in total, 44 PBCRs required the DTA and 3 signed it in a second phase. Discussions between legal officers from PBCRs and the BENCHISTA team regarding the acceptable wording of the DTA continued over a period of 18 months before finalisation of the DTA, leading to a delay in the project’s timeline. For one participating centre a country-specific transparency statement was generated and made publicly available in the project’s website. Another participating centre required the Data Protection Impact Assessment (DPIA) in a format that complied with their specific requirements. The DPIA, evaluates the impact of the processing activity generated within the BENCHISTA Project focusing on the rights and freedoms of the data subjects. The outcome of this assessment was categorized as data processing with a low risk level.

Noticeable differences in interpretation of legislations, laws and required processes were encountered whilst confirming the requirements for participation by each PBCR. For some PBCRs, interpretation of their national laws meant they were not allowed to share patient-level data. These registries reluctantly dropped out of the project as the anticipated work to change this interpretation for sharing this standard dataset was felt to be too complex or not possible. The requirement for local ethical approval, above and beyond sharing of the sponsors’ ethical approval documents, also varied between countries and sometimes between regions within the same country.

After multiple interactions from legal representatives, the DTA was fully executed on 14^th^ November 2022. After final signatures, other PBCRs expressed their interest in participating in the project and submitting data and some others that did not need the signature in the first place but then required it. For these cases the ‘accession document’ included in the Appendix of the DTA was created by the legal officer at INT. This latter document did not permit any further changes to the wording of the DTA.

### 3.3 Survey on data collection/data sharing processes

**Figure 2:**
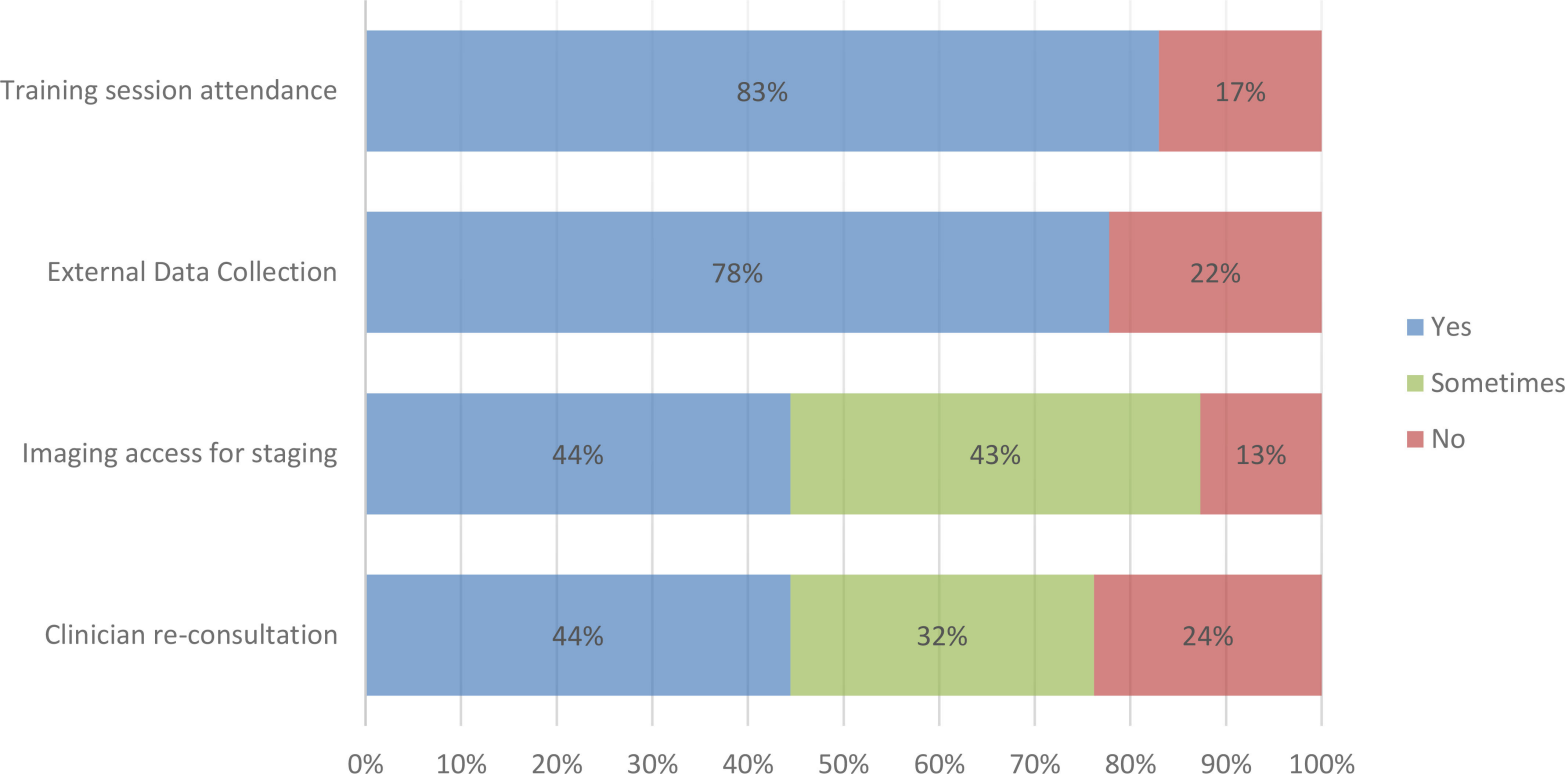
Results of survey on data collection/sharing processes.

The BENCHISTA Project conducted a survey among all participating PBCRs to gather information on available data sources, as well as approaches used for data collection and interpretation. The survey received responses from 63 out of the 67 cancer registries (94%) involved in the project, representing 31 countries (**Table 2** and **Figure 2**).

**Table 2:** Answers to Survey on data collection per country.

Participating Cancer Registry	Online training session attendance	External Data Collection	Possibility to re-consult a clinician	Imaging access for staging
Australia	NO	NO	NO	YES
Belgium	YES	YES	YES	SOMETIMES
Brazil	YES	YES	SOMETIMES	SOMETIMES
Bulgaria	YES	YES	YES	YES
Ontario	NO	YES	YES	YES
Croatia	YES	YES	SOMETIMES	SOMETIMES
Czech Republic (Hospital Brno)	YES	NO	YES	YES
Czech Republic	YES	YES	YES	YES
Denmark	YES	YES	YES	YES
England	YES	YES	NO	SOMETIMES
Estonia	YES	YES	YES	SOMETIMES
France	YES	NO	YES	YES
Germany	YES	NO	SOMETIMES	NO
Greece	YES	YES	SOMETIMES	SOMETIMES
Hungary	YES	YES	YES	SOMETIMES
Ireland	YES	YES	NO	YES
Osaka	YES	YES	YES	NO
Malta	NO	YES	SOMETIMES	YES
The Netherlands	NO	NO	YES	NO
Northern Ireland	YES	YES	SOMETIMES	YES
Norway	YES	YES	SOMETIMES	SOMETIMES
Poland	YES	YES	YES	SOMETIMES
Portugal	YES	YES	YES	YES
Romania	YES	YES	YES	SOMETIMES
Scotland	YES	YES	YES	YES
Slovakia	NO	YES	YES	SOMETIMES
Slovenia	YES	YES	SOMETIMES	YES
Sweden	YES	YES	YES	SOMETIMES
Switzerland	YES	YES	SOMETIMES	SOMETIMES
Wales	YES	YES	YES	YES

Among the respondents, 83% participated in the online training workshops. Additionally, 49 out of 63 respondents (78%) confirmed their ability to collect external data if necessary. In cases where stage ascertainment diagnosis posed difficulties or uncertainties, 28 out of 63 respondents (44%) reported the ability to seek consultation with a clinician. In addition, 20 out of 63 registries (32%) stated that re-consultation was only possible under specific circumstances, such as the availability of clinicians, limited access to clinical records, or depending on the anatomical location of the tumour. For 15 out of 63 registries (24%), clinical re-consultation was not available.

Additionally, 44% of respondents reported having access to individual-patient imaging results for staging purposes, while 13% did not have access to such resources. 43% indicated that they had some access, but not for all cases.

### 3.4 Data standardization and quality assurance

To maximize the efficiency of cancer registry staff time, the project’s quality assurance tool was limited to twelve fictitious cases (two for each tumour type). These cases were designed by the project leaders, discussed among the members of the Project Management Team and the relevant tumour-specific clinical experts, and piloted with a limited number of PBCR staff who had been involved in the design of the training workshops. The cases were then refined and revised for clarity, readability and correctness.

All participating PBCRs were invited to stage these 12 fictitious cases blind to any answers by others. The Project Working Group agreed in advance that a concordance rate of 90% or greater should be aimed for to demonstrate sufficient standardisation.

**Figure 3:**
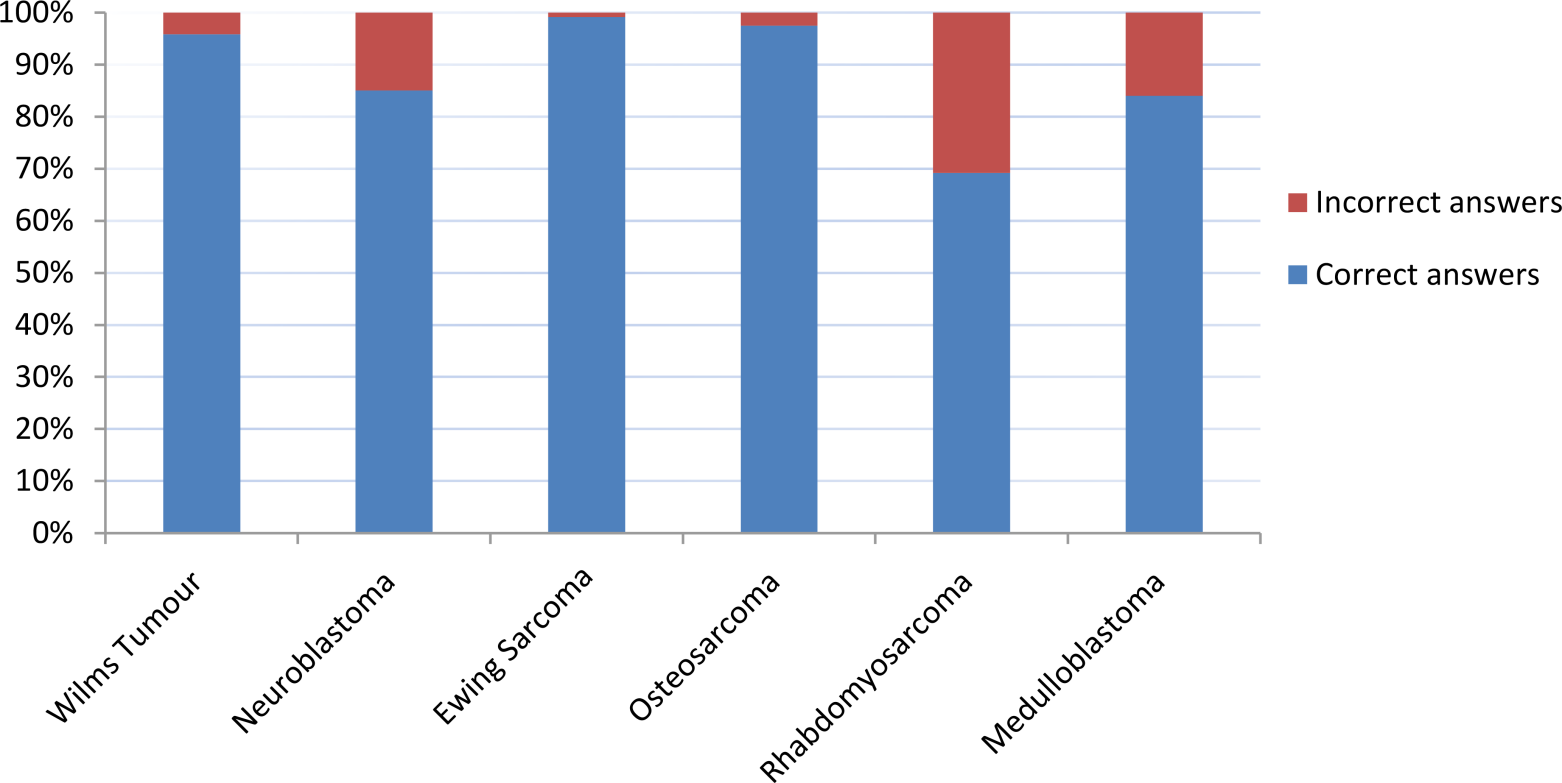
Results of quality assurance tool.

Of the total participating PBCRs (24 out of 29 countries) 75% completed this exercise. The correct answer proportion for the Toronto stage ranged from 70% (rhabdomyosarcoma, Tier 2) to 100% (Wilms tumour), with a median score of 92% (**Figure 3**).

The average correct score varied across registries, ranging from 67% to 100%. The score for rhabdomyosarcoma was lower due to limited correctness in one of the fictitious case exercises (50% correct answers) where assignment of the ‘paranasal’ sinus anatomical site to either favourable or unfavourable category led to a change in the assigned Toronto stage from I to III. For neuroblastoma, the discrepancies were mainly related to variable interpretation of ‘image-defined’ risk factors. For medulloblastoma, there was variable interpretation of whether Tier 2 staging could be applied in the absence of a cerebrospinal fluid (CSF) cytology result. These specific cases highlighted the importance of training and clear definitions in support information such as the CanStaging+ Tool (18, 19) when staging complex cases. The ‘artificial’ nature of the fictitious cases also contributed to discrepancies as registry staff would have access to multiple data sources for cross-verification and to advice from senior colleagues for ‘real’ clinical cases.

As of May 2023, out of the total expected cases, ~98% or 10,504 cases were collected. Information on the stage is complete for 94% of the cases at Tier 1 level and 88% at Tier 2 level for all six tumours combined. Regarding optional variables, completeness is 73% for relapse or progression, while treatment variables such as surgery, chemotherapy, and radiotherapy have a completeness of 83%, 86%, and 81% respectively. NSP have a completeness of 44%. Additionally, cause of death data was reported in 69% of cases.

## 4 Discussion

The BENCHISTA Project has enabled PBCRs from different geographical areas within and beyond Europe to share patient-level data to better understand the factors underlying variation in childhood cancer survival rates. Participation in the project has stimulated their efforts to access the data required to apply the Toronto Guidelines to stage their cases in a standardized way that allows international comparisons. In addition, the feasibility to collect other non-stage prognostic factors and summary information on treatments given, relapse and cause of death has been demonstrated.

The project has focused on achieving consistent participation and compilation of information in line with local or national laws despite legal heterogeneity that led to delays in finalising requirements such as the DTA impacting the project’s timeline. Despite this, the BENCHISTA Project has achieved participation from across most of Europe and with several key international partners to compile detailed information on nearly 11,000 cases of six childhood solid tumours diagnosed in a recent period at a population-level. This is a tremendous ‘proof of principle’ to catalyse continued outcomes research that uses routine healthcare data available to PBCRs. The project has revealed aspects of data access and staging definitions that require further attention if we are to achieve the ultimate aim of truly harmonized data to ensure reliable estimates and survival comparisons.

Several challenges were observed during the DTA generation and sign off by PBCRs. Initially differences in legislation and laws on data sharing/transfer and processing were encountered. Multiple interactions across legal and cancer registry staff were required to reach a consensus on the requirements for data transfer. Additional limiting factors included, different understanding and application of the terms “anonymous” versus “maximally de-personalised” in relation to general data protection regulations, introduction of new data systems that led to delays in finalising cancer incidence data, limited access to clinical data sources and advice for applying TGs, limited registry workforce capacity for data collection/staging tasks in specific timelines, administrative changes or high turn-over leading to delays. Some of these difficulties were more noticeable in countries with regional rather than national coverage, where the interpretation of General Data Protection Regulations varied between regions.

Nevertheless, data available to PBCRs has generally increased over time, with direct data feeds from clinical reporting (histopathology, imaging, treatments etc) permissible in many jurisdictions. In spite of this, access to the detailed information and clinical support required to apply Toronto staging at Tier 2, together with non-stage prognostic variables, remains very variable.

Considering the results from the survey on data collection, there is noticeable variation in the access to data sources at an individual patient-level and available clinical support. This may impact the application of TG in some cases and highlights the importance of understanding current modalities for data collection and standardised parameters for staging by PBCRs.

The quality assurance tool aimed to assess how standardised the collection of TG is across the PBCRs. This exercise highlighted challenges related to differences in terminology, risk group definitions and access to required clinical information to complete TG accurately. Some examples include the availability of CSF cytology results for medulloblastoma, image-defined risk factors for neuroblastoma and differences in interpretation/classification of favourable and unfavourable anatomical sites for rhabdomyosarcoma. These were discussed among the Independent Advisory Board and other members of the project leading to further conversations with TG leaders, clinicians, cancer registry staff and researchers to improve the understanding of key clinical parameters to improve staging and therefore healthcare data research. Recommendations from this project’s experience are already being considered for inclusion in the next revision of the CanStaging+ Tool (5, 18).

Additionally, a key point discussed among the project’s team focused on the importance of standardization of the definition of *metastasis*, particularly in relation to lung nodules (for Wilms Tumour and all three sarcomas) and how it requires further attention. PBCR staff rely on the interpretation provided in the imaging reports or by the clinician providing the stage information. These are inherently variable (in their definitions of metastasis) and could benefit from a move towards standardised structured reporting.

Considering previous challenges and to enhance communication channels, project-specific resources were created to ensure consistent interaction with PBCRs; these include newsletters, social media and web platforms, on-line meetings and active participation on scientific conferences. However, this might not be sustainable beyond research-funded activities. In spite of this, the project has provided valuable insights to demonstrate the viability of the general approach of PBCRs collecting and sharing patient-level routine health care data. This approach not only paves the way for continuous benchmarking of stage distribution at diagnosis and survival by stage, but also serves as the foundation for population-level outcomes research in cancers with different prognosis. For example, in tumour-types and subgroups with overall survival rates in excess of 90%, implementation and funding of prospective clinical trials is increasingly challenging. Hence, the ability to design prospective studies that can use the capabilities of PBCRs to collect additional non-stage prognostic variables, offers an efficient mechanism to monitor population-level survival rates for clinically defined subgroups for whom there are no open interventional clinical trials but who are treated according to nationally agreed clinical practice guidelines.

The BENCHISTA Project represents an opportunity to understand reasons for international variation in overall survival for childhood cancer at a population-level by enhancing the collaboration with PBCRs and stimulating their ability to use TG in childhood cancer cases in a standardized way. Data harmonization also requires strengthened relationships with clinicians, medical sources, the European Network of Cancer Registries and other stakeholders to ensure cancer data recorded in registries are high-quality, comprehensive and accurate empowering the PBCRs to routinely collect TG for future benchmarking research leading to outcome improvement.

## Data availability statement

The original contributions presented in the study are included in the article/supplementary material. Further inquiries can be directed to the corresponding author.

## Ethics statement

In compliance with institutional and legal requirements, the project was granted with ethical approvals from UCL and INT.

## Author contributions

KP-J is the principal investigator of the BENCHISTA Project, and GG is a co-investigator, both with equal contribution and senior authorship. AL-C, LB and FD have equal contribution as joint first authors and contributed to writing – original draft and editing. FD and LB contributed also to formal analysis. KP-J, GG, AL-C, LB, and FD contributed also to conceptualization, project administration and methodology. The Project Management Team (PMT) members supported in the discussion of the methods and interpretation of the results. The Project Working Group (PWG) contributed to data collection, interpretation and discussion of the results. All authors approved the submitted version.

## BENCHISTA Project Working Group

For a full list of the international members of the BENCHISTA Project Working Group, see Appendix 3.

## Appendix 3 The BENCHISTA Project Working Group:

**Australia:** Australian Childhood Cancer Registry: Joanne Aitken, Leisa O’Neill;

**Austria:** Austrian Cancer Registry: Monika Hackl, Ruth Ladenstein;

**Belgium:** Belgian Cancer Registry: Elizabeth Van Eycken, Nancy Van Damme;

**Boston, MA (USA):** Dana-Farber Cancer Institute: Lindsay Frazier;

**Brasil:** Cancer Registry Representatives: Beatriz De Camargo, Marceli de Oliveira Santos;

**Bulgaria:** Bulgarian Cancer Registry: Zdravka Valerianova, Dobrin Konstantinov;

**Canada:** OOntario Children Cancer Registry_POGO: Sumit Gupta, Jason D Pole;

**Croatia:** Croatian Cancer Registry: Mario Sekerija;

**Czech Republic:** Czech National Cancer Registry: Jan Stary, Jaroslav Sterba;

**Denmark:** Danish Childhood Cancer Registry and Department of Pediatric Oncology: Lisa L. Hjalgrim (PMT Member); Childhood Cancer Research Group, Danish Cancer Institute, Copenhagen, Denmark & Department of Clinical Medicine, Faculty of Health, Aarhus University and University Hospital, Aarhus, Denmark: Jeanette Falck Winther;

**Estonia:** Estonia National Institute for Health Development: Keiu Paapsi;

**France:** French National Registry of Childhood Cancer - *Solid tumours:* Brigitte Lacour, Emmanuel Desandes; *Hematopoietic Malignancies:* Jacqueline Clavel, Claire Poulalhon;

**Germany:** German Childhood Cancer Registry, Mainz: Claudia Spix, Meike Ressing;

**Greece:** Greek Nationwide Registry for Childhood Hematological Malignancies and Solid Tumours (NARECHEMST): Eleni T Petridou, Evdoxia Bouka;

**Hungary:** Hungarian Child Cancer Registry: Zsuzsanna Jakab (PMT Member), Miklos Garami;

**Italy:**

 Basilicata Cancer Registry: Rocco Galasso;

 Bergamo Cancer Registry: Giuseppe Sampietro;

 Campania Childhood Cancer Registry: Francesco Vetrano, Marcella Sessa;

 Childhood Cancer Registry of Piedmont: Milena M Maule, Carlotta Sacerdote;

 Cremona & Mantova Cancer Registry: Paola Ballotari;

 Friuli Venezia Giulia Cancer Registry, CRO Aviano National Cancer Institute: Emilia De Santis;

 Integrated Cancer Registry CT-ME-EN: Margherita Ferrante, Rosalia Ragusa;

 Innovation, Research and Teaching Service (SABES-ASDAA), Teaching Hospi tal of the Paracelsus Medizinischen Privatuniversität, Bolzano-Bozen, Italy: Michael Mian;

 Liguria Cancer Registry, Ospedale Policlinico San Martino IRCCS: Luca Boni;

 Monza-Brianza Cancer Registry: Magda Rognoni;

 Palermo Province Cancer Registry: Rosalba Amodio;

 Pavia Cancer Registry: Lorenza Boschetti;

 Puglia Cancer Registry: Francesco Cuccaro, Danila Bruno;

 Registro tumori ATS della Città metropolitana di Milano: Antonio G Russo, Federico Gervasi;

 Registro Tumori ATS Insubria: Maria L Gambino;

 Registro tumori dell’Emilia-Romagna, Unità di Piacenza: Elisabetta Borciani;

 Registro tumori dell’Emilia-Romagna, Unità di Parma: Maria Michiara;

 Registro tumori dell’Emilia-Romagna, Unità di Reggio Emilia: Lucia Mangone;

 Registro tumori dell'Emilia-Romagna, Unità di Modena: Gianbattista Spagnoli;

 Registro tumori dell'Emilia-Romagna, Unità di Ferrara: Stefano Ferretti;

 Registro tumori dell'Emilia-Romagna, Unità della Romagna, IRCCS IRST Meldola: Fabio Falcini;

 Registro Tumori di Ragusa e Caltanissetta: Eugenia Spata;

 Registro Tumori Regione Marche: Sonia Manasse, Paola Coccia;

 Siracusa Province Cancer Registry: Francesca Bella;

 Toscana Cancer Registry: Adele Caldarella, Teresa Intrieri;

 Trapani Cancer Registry: Tiziana Scuderi;

 Trento Cancer Registry (Trento CR), Servizio Epidemiologia Clinica e Valutativa, APSS Trento: Roberto V Rizzello;

 Veneto Cancer Registry, Regional Epidemiological Service, Azienda Zero, Padova, Italy: Massimo Rugge, Stefano Guzzinati;

**Ireland:** Ireland Cancer Registry: Deirdre Murray;

**Japan:** NNational Cancer Center: Tomohiro Matsuda; Osaka CR: Kayo Nakata;

**Malta:** Malta National Cancer Registry, Health Information and Research: Miriam J Azzopardi;

**Norway:** Norwegian Cancer Registry: Tom Børge Johannesen, Aina H Dahlen; Bernward Zeller (PMT Member);

**Poland:** Polish Childhood Cancer Clinical Database provided by the Polish Society of Paediatric Oncology and Haematology (PSPOH) - Prof Jerzy Kowalczyk, Medical University of Lublin and Prof Anna Raciborska, Department of Oncology and Surgical Oncology for Children and Youth, Institute of Mother and Child.

**Portugal:** Portuguese Pediatric Cancer Registry: Ana M Ferreira, Gabriela Caldas;

**Romania:** Romanian Child Cancer Registry: Mihaela Bucurenci, Dana Coza;

**Slovenia:**Cancer Registry of Republic of Slovenia: Vesna Zadnik;

**Spain:**

 Basque Country, Euskadi-CIBERESP Cancer Registry: Arantza Lopez de Munain;

 Childhood and Adolescents CR - CISCV: Fernando Almela-Vich, Nieves Fuster-Camarena;

 Girona CR, CIBERESP, ICO, IDIBGI: Rafael Marcos-Gragera;

 Granada CR, EASP, CIBERESP, ibs.GRANADA, UGR: Maria José Sanchez;

 Madrid Childhood Cancer Registry: Aragonés, Raquel López;

 Murcia Cancer Registry, CIBERESP, IMIB-Arrixaca: Maria Dolores Chirlaque;

 Navarra Cancer Registry, ISPLN, CIBERESP, IdiSNA: Marcela Guevara; Peris-Bonet, Adela Cañete Nieto (PMT Member);

 Tarragona Cancer Registry, HUSJR, IISPV: Marià Carulla;

**Sweden:** Swedish Childhood Cancer Registry – SCCR, Karolinska Institute, Stockholm, Sweden: Päivi Lähteenmäki;

**Switzerland:** Childhood Cancer Registry, Institute of Social and Preventive Medicine, University of Bern, Switzerland: Claudia E Kuehni, Shelagh M Redmond;

**The Netherlands:** The Netherlands Cancer Registry: Otto Visser, Henrike Karim-Kos;

**United Kingdom:**

 England: National Disease Registration Service, Transformation Directorate, NHS England: Lucy Irvine, Paul Stacey, Charles Stiller (PMT Member);

 Northern Ireland Cancer Registry : Anna Gavin, Deirdre Fitzpatrick;

 Scotland: Scottish Cancer Registry: David S Morrison; Karen Smith;

 Welsh Cancer Intelligence and Surveillance Unit, Public Health Wales: Dyfed Wyn Huws, Janet Warlow;

**Clinical Lead Experts:**

**Ewing Sarcoma:** Sandra Strauss - University College London Hospital (UCLH), London, England, UK.

**Medulloblastoma:** Simon Bailey - Great North Children’s Hospital, Newcastle upon Tyne, England, UK.

**Neuroblastoma:**Adela Cañete Nieto - Hospital UiP La Fe, Paediatric Oncology and Hematology Unit, Valencia, Spain.

**Osteosarcoma:** Nathalie Gaspar - Gustave Roussy Cancer Campus, Villejuif, France.

**Rhabdomyosarcoma:** Lisa L. Hjalgrim - University of Copenhagen, Rigshospitalet, Department of Pediatrics and Adolescent Medicine, Copenhagen, Denmark.

**Wilms Tumour:** Filippo Spreafico - IRCCS National Cancer Institute, Milan, Italy.

Angela Polanco (Patient and Public Involvement and Engagement – PPIE Representative);

Riccardo Capocaccia (Epidemiologia & Prevenzione, Italy);

Andrea Di Cataldo (Università degli Studi di Catania, Italy);

Meric Klein (Belgian Cancer Registry – Training Workshops Lead).
