# Corrigendum: Cancer data quality and harmonization in Europe: The experience of the BENCHISTA Project – International Benchmarking of Childhood Cancer Survival by Stage

**DOI:** 10.3389/fonc.2024.1397101

**Published:** 2024-03-28

**Authors:** Angela Lopez-Cortes, Fabio Didonè, Laura Botta, Lisa L. Hjalgrim, Zsuzsanna Jakab, Adela Cañete Nieto, Charles Stiller, Bernward Zeller, Gemma Gatta, Kathy Pritchard-Jones, The BENCHISTA Project Working Group

**Affiliations:** ^1^ University College London (UCL) Great Ormond Street Institute of Child Health, Developmental Biology & Cancer Research Department, London, United Kingdom; ^2^ Fondazione IRCCS “Istituto Nazionale dei Tumori di Milano” (INT), Department of Evaluative Epidemiology, Milan, Italy; ^3^ BENCHISTA Project Management Team, London, United Kingdom

**Keywords:** childhood cancer, population-based, cancer registry, Toronto staging, diagnosis, survival, data quality, data harmonization

In the published article, there was an error in Supplementary **Appendix (Number 3)**. The Project Working Group was displayed according to initial known information. A concern related to the Polish representative affiliation was raised after submission. After internal review and discussion with the relevant involved parties, an agreement on the amendment to the name/affiliation of the Polish representative and clarification to the source of data collection was reached. Furthermore, other changes to the Appendix 3 were incorporated as these were communicated by other representatives from the Project Working Group after the article deadline/submission.

The appendix in the published article was displayed as:

**Appendix 3**

**The BENCHISTA Project Working GroupƗ:**

**Australia:** Australian Cancer Registry (CR): Joanne Aitken, Leisa O’Neill;

**Austria:** Austrian CR: Monika Hackl, Ruth Ladenstein;

**Belgium:** Belgian CR: Elizabeth Van Eycken, Nancy Van Damme;

**Boston, MA (USA):** Dana-Farber Cancer Institute: Lindsay Frazier;

**Brasil:** CR Representatives: Beatriz De Camargo, Marceli de Oliveira Santos;

**Bulgaria:** Bulgarian CR: Zdravka Valerianova, Dobrin Konstantinov;

**Canada:** Ontario Children CR_POGO: Sumit Gupta, Jason D Pole;

**Croatia:** Croatian CR: Mario Sekerija;

**Czech Republic:** Czech National CR: Jan Stary, Jaroslav Sterba;

**Denmark:** Danish Childhood Cancer Registry and Department of Pediatric Oncology: Lisa L. Hjalgrim (PMT Member); Danish Cancer Society: Jeanette F Winther;

**Estonia:** Estonia National Institute for Health Development: Keiu Paapsi;

**France:** French National Registry of Childhood Cancer - Solid tumours: Brigitte Lacour, Emmanuel Desandes; Hematopoietic Malignancies: Jacqueline Clavel, Claire Poulalhon;

**Germany:** German Childhood CR, Mainz: Friederike Erdmann, Claudia Spix;

**Greece:** Greek Nationwide Registry for Childhood Hematological Malignancies and Solid Tumours (NARECHEM-ST): Eleni T Petridou, Evdoxia Bouka;

**Hungary:** Hungarian Child CR: Zsuzsanna Jakab (PMT Member);

**Italy:**

Alto Adige CR: Michael Mian;

Basilicata CR: Rocco Galasso;

Bergamo CR: Giuseppe Sampietro;

Campania Childhood CR: Francesco Vetrano, Marcella Sessa;

Childhood Cancer Registry of Piedmont: Milena M Maule, Carlotta Sacerdote;

Cremona & Mantova CR: Paola Ballotari;

Friuli Venezia Giulia CR, CRO Aviano National Cancer Institute: Luigino Dal Maso; Integrated Cancer Registry CT-ME-EN: Margherita Ferrante, Rosalia Ragusa;

Liguria CR, Ospedale Policlinico San Martino IRCCS: Luca Boni;

Monza-Brianza CR: Magda Rognoni;

Palermo CR: Rosalba Amodio;

Pavia CR: Lorenza Boschetti;

Puglia CR: Francesco Cuccaro, Danila Bruno;

Registro tumori ATS della Città metropolitana di Milano: Antonio G Russo, Federico Gervasi; Registro Tumori ATS Insubria: Maria L Gambino;

Registro tumori dell’Emilia-Romagna, Unità di Piacenza: Elisabetta Borciani;

Registro tumori dell’Emilia-Romagna, Unità di Parma: Maria Michiara;

Registro tumori dell’Emilia-Romagna, Unità di Reggio Emilia: Lucia Mangone;

Registro tumori dell’Emilia-Romagna, Unità di Modena: Gianbattista Spagnoli;

Registro tumori dell’Emilia-Romagna, Unità di Ferrara: Stefano Ferretti;

Registro tumori dell’Emilia-Romagna, Unità della Romagna, IRCCS IRST Meldola: Fabio Falcini;

Registro Tumori di Ragusa e Caltanissetta: Eugenia Spata;

Registro Tumori Regione Marche: Sonia Manasse, Paola Coccia;

Siracusa Province CR: Francesca Bella;

Toscana CR: Adele Caldarella, Teresa Intrieri;

Trapani CR: Tiziana Scuderi;

Trento Cancer Registry (Trento CR), Servizio Epidemiologia Clinica e Valutativa, APSS Trento: Roberto V Rizzello;

Veneto CR: Massimo Rugge, Stefano Guzzinati;

**Ireland:** Ireland CR: Deirdre Murray;

**Japan:** National Cancer Center: Tomohiro Matsuda; Osaka CR: Kayo Nakata;

**Malta:** Malta National Cancer Registry, Health Information and Research: Miriam J Azzopardi;

**Norway:** Norwegian CR: Tom Børge Johannesen, Aina H Dahlen; Bernward Zeller (PMT Member);

**Poland:** Polish Childhood Cancer Registry, Medical University of Lublin: Jerzy Kowalczyk; Polish Institute of Mother and Child: Monika Jedrzejczyk;

**Portugal:** Portuguese Pediatric CR: Ana M Ferreira, Gabriela Caldas;

**Romania:** Romanian Child CR: Mihaela Bucurenci, Dana Coza;

**Slovenia:** Cancer Registry of Republic of Slovenia: Vesna Zadnik;

**Spain:**

Basque Country, Euskadi-CIBERESP CR: Arantza Lopez de Munain;

Childhood and Adolescents CR - CISCV: Fernando Almela-Vich, Nieves Fuster-Camarena; Girona CR, CIBERESP, ICO, IDIBGI: Rafael Marcos-Gragera;

Granada CR, EASP, CIBERESP, ibs.GRANADA, UGR: Maria José Sanchez;

Madrid Childhood CR: Nuria Aragones, Raquel Lopez;

Murcia CR, CIBERESP, IMIB-Arrixaca: Maria Dolores Chirlaque;

Navarra CR, CIBERESP, IdiSNA: Marcela Guevara Eslava;

Spain RETI-SEHOP: Elena Pardo, Rafael Peris-Bonet, Adela Cañete Nieto (PMT Member); Tarragona CR: Jaume Galceran;

**Sweden:** Swedish Childhood Cancer Registry - SCCR: Päivi Lähteenmäki;

**Switzerland:** Childhood Switzerland CR: Claudia E Kuehni, Shelagh M Redmond;

The **Netherlands:** The Netherlands CR: Otto Visser, Henrike Karim-Kos;

**United Kingdom:**

England: National Disease Registration Service, Transformation Directorate, NHS England: Lucy Irvine, Paul Stacey, Charles Stiller (PMT Member);

Northern Ireland CR: Anna Gavin, Deirdre Fitzpatrick;

Scotland: Scottish CR: David S Morrison; Karen Smith;

Welsh Cancer Intelligence and Surveillance Unit - WCISU: Dyfed Wyn Huws, Janet Warlow;

**Clinical Lead Experts:**

Ewing Sarcoma: Sandra Strauss - University College London Hospital (UCLH), London, England, UK.

Medulloblastoma: Simon Bailey - Great North Childrens Hospital, Newcastle upon Tyne, England, UK.

Neuroblastoma: Adela Canete Nieto -Hospital UiPLaFe, Paediatric Oncology and Hematology Unit, Valencia, Spain.

Osteosarcoma: Nathalie Gaspar - Gustave Roussy Cancer Campus, Villejuif, France. Rhabdomyosarcoma: Lisa L. Hjalgrim - University of Copenhagen, Rigshospitalet, Department of Pediatrics and Adolescent Medicine, Copenhagen, Denmark.

Wilms Tumour: Filippo Spreafico - IRCCS National Cancer Institute, Milan, Italy.

Patient and Public Involvement and Engagement – PPIE representative: Angela Polanco;

Epidemiologia & Prevenzione, Italy: Riccardo Capocaccia;

Università degli Studi di Catania, Italy: Andrea Di Cataldo;

Belgian Cancer Registry: Meric Klein.

The correct material statement appears below:

**Appendix 3**

**The BENCHISTA Project Working GroupƗ**

**Australia:** Australian Childhood Cancer Registry: Joanne Aitken, Leisa O’Neill;

**Austria:** Austrian Cancer Registry: Monika Hackl, Ruth Ladenstein;

**Belgium:** Belgian Cancer Registry: Elizabeth Van Eycken, Nancy Van Damme;

**Boston, MA (USA):** Dana-Farber Cancer Institute: Lindsay Frazier;

**Brasil:** Cancer Registry Representatives: Beatriz De Camargo, Marceli de Oliveira Santos;

**Bulgaria:** Bulgarian Cancer Registry: Zdravka Valerianova, Dobrin Konstantinov;

**Canada:** Ontario Children Cancer Registry _ POGO: Sumit Gupta, Jason D Pole;

**Croatia:** Croatian Cancer Registry: Mario Sekerija;

**Czech Republic:** Czech National Cancer Registry: Jan Stary, Jaroslav Sterba;

**Denmark:** Danish Childhood Cancer Registry and Department of Pediatric Oncology: Lisa L. Hjalgrim (PMT Member); Childhood Cancer Research Group, Danish Cancer Institute, Copenhagen, Denmark & Department of Clinical Medicine, Faculty of Health, Aarhus University and University Hospital, Aarhus, Denmark: Jeanette Falck Winther;

**Estonia:** Estonia National Institute for Health Development: Keiu Paapsi;

**France:** French National Registry of Childhood Cancer - *Solid tumours:* Brigitte Lacour, Emmanuel Desandes; *Hematopoietic Malignancies:* Jacqueline Clavel, Claire Poulalhon;

**Germany:** German Childhood Cancer Registry, Mainz: Claudia Spix, Meike Ressing;

**Greece:** Greek Nationwide Registry for Childhood Hematological Malignancies and Solid Tumours (NARECHEM-ST): Eleni T Petridou, Evdoxia Bouka;

**Hungary:** Hungarian Child Cancer Registry: Zsuzsanna Jakab (PMT Member), Miklos Garami;

**Italy:**

Basilicata Cancer Registry: Rocco Galasso;

Bergamo Cancer Registry: Giuseppe Sampietro;

Campania Childhood Cancer Registry: Francesco Vetrano, Marcella Sessa;

Childhood Cancer Registry of Piedmont: Milena M Maule, Carlotta Sacerdote;

Cremona & Mantova Cancer Registry: Paola Ballotari;

Friuli Venezia Giulia Cancer Registry, CRO Aviano National Cancer Institute: Emilia De Santis;

Integrated Cancer Registry CT-ME-EN: Margherita Ferrante, Rosalia Ragusa;

Innovation, Research and Teaching Service (SABES-ASDAA), Teaching Hospital of the Paracelsus Medizinischen Privatuniversität, Bolzano-Bozen, Italy: Michael Mian;

Liguria Cancer Registry, Ospedale Policlinico San Martino IRCCS: Luca Boni;

Monza-Brianza Cancer Registry: Magda Rognoni;

Palermo Province Cancer Registry: Rosalba Amodio;

Pavia Cancer Registry: Lorenza Boschetti;

Puglia Cancer Registry: Francesco Cuccaro, Danila Bruno;

Registro tumori ATS della Città metropolitana di Milano: Antonio G Russo, Federico Gervasi;

Registro Tumori ATS Insubria: Maria L Gambino;

Registro tumori dell’Emilia-Romagna, Unità di Piacenza: Elisabetta Borciani;

Registro tumori dell’Emilia-Romagna, Unità di Parma: Maria Michiara;

Registro tumori dell’Emilia-Romagna, Unità di Reggio Emilia: Lucia Mangone;

Registro tumori dell’Emilia-Romagna, Unità di Modena: Gianbattista Spagnoli;

Registro tumori dell’Emilia-Romagna, Unità di Ferrara: Stefano Ferretti;

Registro tumori dell’Emilia-Romagna, Unità della Romagna, IRCCS IRST Meldola: Fabio Falcini;

Registro Tumori di Ragusa e Caltanissetta: Eugenia Spata;

Registro Tumori Regione Marche: Sonia Manasse, Paola Coccia;

Siracusa Province Cancer Registry: Francesca Bella;

Toscana Cancer Registry: Adele Caldarella, Teresa Intrieri;

Trapani Cancer Registry: Tiziana Scuderi;

Trento Cancer Registry (Trento CR), Servizio Epidemiologia Clinica e Valutativa, APSS Trento: Roberto V Rizzello;

Veneto Cancer Registry, Regional Epidemiological Service, Azienda Zero, Padova, Italy: Massimo Rugge, Stefano Guzzinati;

**Ireland:** Ireland Cancer Registry: Deirdre Murray;

**Japan:** National Cancer Center: Tomohiro Matsuda; Osaka CR: Kayo Nakata;

**Malta:** Malta National Cancer Registry, Health Information and Research: Miriam J Azzopardi;

**Norway:** Norwegian Cancer Registry: Tom Børge Johannesen, Aina H Dahlen; Bernward Zeller (PMT Member);

**Poland:** Polish Childhood Cancer Clinical Database provided by the Polish Society of Paediatric Oncology and Haematology (PSPOH) - Prof Jerzy Kowalczyk, Medical University of Lublin and Prof Anna Raciborska, Department of Oncology and Surgical Oncology for Children and Youth, Institute of Mother and Child.

**Portugal:** Portuguese Pediatric Cancer Registry: Ana M Ferreira, Gabriela Caldas;

**Romania:** Romanian Child Cancer Registry: Mihaela Bucurenci, Dana Coza;

**Slovenia:** Cancer Registry of Republic of Slovenia: Vesna Zadnik;

**Spain:**

Basque Country, Euskadi-CIBERESP Cancer Registry: Arantza Lopez de Munain;

Childhood and Adolescents Cancer Registry - CISCV: Fernando Almela-Vich, Nieves Fuster-Camarena;

Girona Cancer Registry, CIBERESP, ICO, IDIBGI: Rafael Marcos-Gragera;

Granada Cancer Registry, EASP, CIBERESP, ibs.GRANADA, UGR: Maria José Sanchez;

Madrid Childhood Cancer Registry: Nuria Aragonés, Raquel López;

Murcia Cancer Registry, CIBERESP, IMIB-Arrixaca: Maria Dolores Chirlaque;

Navarra Cancer Registry, ISPLN, CIBERESP, IdiSNA: Marcela Guevara;

Spanish Registry of Childhood Tumours (RETI SEHOP), University of Valencia, Faculty of Medicine: Elena Pardo, Rafael Peris-Bonet, Adela Cañete Nieto (PMT Member);

Tarragona Cancer Registry, HUSJR, IISPV: Marià Carulla;

**Sweden:** Swedish Childhood Cancer Registry – SCCR, Karolinska Institute, Stockholm, Sweden: Päivi Lähteenmäki;

**Switzerland:** Childhood Cancer Registry, Institute of Social and Preventive Medicine, University of Bern, Switzerland: Claudia E Kuehni, Shelagh M Redmond;

**The Netherlands:** The Netherlands Cancer Registry: Otto Visser, Henrike Karim-Kos;

**United Kingdom:**

England: National Disease Registration Service, Transformation Directorate, NHS England: Lucy Irvine, Paul Stacey, Charles Stiller (PMT Member);

Northern Ireland Cancer Registry: Anna Gavin, Deirdre Fitzpatrick;

Scotland: Scottish Cancer Registry: David S Morrison; Karen Smith;

Welsh Cancer Intelligence and Surveillance Unit, Public Health Wales: Dyfed Wyn Huws, Janet Warlow;

**Clinical Lead Experts:**

**Ewing Sarcoma:** Sandra Strauss - University College London Hospital (UCLH), London, England, UK.

**Medulloblastoma:** Simon Bailey - Great North Children’s Hospital, Newcastle upon Tyne, England, UK.

**Neuroblastoma:** Adela Cañete Nieto - Hospital UiP La Fe, Paediatric Oncology and Hematology Unit, Valencia, Spain.

**Osteosarcoma:** Nathalie Gaspar - Gustave Roussy Cancer Campus, Villejuif, France.

**Rhabdomyosarcoma:** Lisa L. Hjalgrim - University of Copenhagen, Rigshospitalet, Department of Pediatrics and Adolescent Medicine, Copenhagen, Denmark.

**Wilms Tumour:** Filippo Spreafico - IRCCS National Cancer Institute, Milan, Italy.

Angela Polanco (Patient and Public Involvement and Engagement – PPIE Representative);

Riccardo Capocaccia (Epidemiologia & Prevenzione, Italy);

Andrea Di Cataldo (Università degli Studi di Catania, Italy);

Meric Klein (Belgian Cancer Registry – Training Workshops Lead).

In the published article, the first paragraph is correct. A phrase is required to be added in the first paragraph of the material and methods section to clarify that not all of the population-based cancer registries that contributed data to the project are constituted in the same way.

A correction has been made to **Materials and Methods**, at end of first paragraph.

This sentence previously stated:

“All European population-based cancer registries (PBCRs) included in the EUROCARE studies were invited to participate in the BENCHISTA Project. Additionally other non-European PBCRs from Australia, Canada, Brazil, and Japan confirmed their contribution to the project. A great number of PBCRs are checked for quality indicators by the International Agency for Research on Cancer (IARC) based in four dimensions of quality: comparability, validity, timeliness, and completeness (16, 17).”

The corrected sentence appears below:

“All European population-based cancer registries (PBCRs) included in the EUROCARE studies were invited to participate in the BENCHISTA Project. Additionally, other non-European PBCRs from Australia, Canada, Brazil, and Japan confirmed their contribution to the project. A great number of PBCRs are checked for quality indicators by the International Agency for Research on Cancer (IARC) based on four dimensions of quality: comparability, validity, timeliness, and completeness (16, 17). Not all PBCRs have a government mandate. Some are coordinated by the National Society for Paediatric Haematology-Oncology and/or register all cases diagnosed at all hospitals authorised for childhood cancer treatment in the relevant country, with the aim of achieving population coverage.”

The authors apologize for these errors and state that this does not change the scientific conclusions of the article in any way. The original article has been updated.

